# Characterization of Pyruvate Uptake in *Escherichia coli* K-12

**DOI:** 10.1371/journal.pone.0067125

**Published:** 2013-06-20

**Authors:** Jens Kreth, Joseph W. Lengeler, Knut Jahreis

**Affiliations:** 1 Department of Microbiology and Immunology, University of Oklahoma Health Sciences Center, Oklahoma City, Oklahoma, United States of America; 2 Arbeitsgruppe Genetik, Fachbereich Biologie/Chemie, Universität Osnabrück, Osnabrück, Germany; University of Groningen, The Netherlands

## Abstract

The monocarboxylate pyruvate is an important metabolite and can serve as sole carbon source for *Escherichia coli*. Although specific pyruvate transporters have been identified in two bacterial species, pyruvate transport is not well understood in *E. coli*. In the present study, pyruvate transport was investigated under different growth conditions. The transport of pyruvate shows specific activities depending on the growth substrate used as sole carbon source, suggesting the existence of at least two systems for pyruvate uptake: i) one inducible system and probably highly specific for pyruvate and ii) one system active under non-induced conditions. Using the toxic pyruvate analog 3-fluoropyruvate, a mutant was isolated unable to grow on and transport pyruvate. Further investigation revealed that a revertant selected for growth on pyruvate regained the inducible pyruvate transport activity. Characterization of pyruvate excretion showed that the pyruvate transport negative mutant accumulated pyruvate in the growth medium suggesting an additional transport system for pyruvate excretion. The here presented data give valuable insight into the pyruvate metabolism and transport of *E. coli* suggesting the presence of at least two uptake systems and one excretion system to balance the intracellular level of pyruvate.

## Introduction

The monocarboxylic acid pyruvate plays an important role in the metabolism of *Escherichia coli* and other enteric bacteria. Together with phosphoenolpyruvate (PEP), it forms the center of the pyruvate-node, a group of metabolites and enzymes located at the interface of carbon catabolism, energy metabolism and anabolism, with a major role in inducer exclusion and carbon catabolite repression [1]. Pyruvate is the terminal product from glycolysis and enters the TCA cycle under aerobic growth conditions. During anaerobic growth, it is the precursor for the generation of heterolactic fermentation products like L-lactate, acetate, ethanol, and others [2].

Pyruvate can be used as sole carbon source. It can also be excreted and reused under certain growth conditions [3]. However, knowledge on pyruvate specific uptake and excretion systems in *E. coli* is scarce. Kornberg and Smith [4] first described the isolation of a phosphoenolpyruvate synthase (*ppsA*) deficient mutant with restored enzymatic activity unable to grow on pyruvate as sole carbon source. The corresponding mutation was mapped near 64 min in a gene locus designated Usp (for *u*ptake *s*pecific for *p*yruvate). The Usp negative mutant was still able to grow on L-lactate. This seems to indicate the existence of at least two different systems for the uptake of the monocarboxylic acids pyruvate and l-lactate. The result contradicts Matin and Konings [5] who, based on transport and competition experiments with vesicles, predicted a common transporter for these mono-carboxylates. A specific permease (gene *lldP*) for L-lactate uptake was described later [6]. The substrate specificity of this permease included besides L-lactate only two other 2-hydroxy-monocarboxilic acids, D-lactate and glycolate, although the corresponding *lld*- operon is induced by L-lactate and pyruvate [6], [7].

Using transport tests, Lang *et al.* [8] were able to measure a pyruvate uptake activity in glycerol grown cells of *E. coli* K-12 with specificity for pyruvate (K_M_ 15 µM), and its analogues 3-bromo-pyruvate (K_i_ 25 µM), 3-fluoropyruvate, and pyruvic acid methyl ester. The system involved lacked detectable affinity for l-lactate. Respiratory chain poisons and uncouplers inhibited uptake, and it required an artificial electron donor system in vesicles, while arsenate was not a specific inhibitor. Pyruvate uptake has also been claimed to be sensitive to osmotic shock [8].

Here we report the isolation and characterization of mutants unable to transport pyruvate. Based on direct transport tests and a genetic analysis of the various mutants, two uptake systems for pyruvate could be identified and characterized further. We also give evidence that a third system is involved in the excretion of pyruvate. To the best of our knowledge this is the first report for more than one pyruvate transport system in *E. coli* K-12.

## Materials and Methods

### Chemicals

Sodium-3-fluoropyruvate (3-FP) was purchased from SIGMA-ALDRICH Chemie GmbH (Seelze, Germany), [1-^14^C] pyruvate was from Amersham Life Technologies (Freiburg, Germany). All other chemicals were of commercially available analytical grades.

### Bacterial strains, plasmids and growth conditions

The *E. coli* K-12 and wild type strains used in this study are listed in Table 1. Strains were grown in phosphate-buffered minimal medium as described previously [9], or in Lennox broth without glucose and calcium ions (LB). Carbohydrates L-lactate, pyruvate, D-glucitol, gluconate, succinate and glycerol were dissolved in deionized water, sterilized by filtration and added to 0.2% final concentration. Growth under potassium limiting conditions was done in phosphate buffered minimal medium as described [10]. Growth was performed at 37°C under vigorous shaking.

**Table 1:** Bacterial strains and plasmids.

Strains/Plasmids	Relevant Characteristics	Reference or Source
**Strains**		
CA8000	Hfr H Prv^+^	[14]
EC3132	Prv^+^	[11]
LAB65	*nagA kdp*::Tn*10* Tet^r^	unpublished
LJ110	W3110 Fnr^+^ Prv^+^	[13]
LJK3	Prv^−^, FP^r^	This study
LJK3R1, LJK3R2	Prv^+^ (partially restored Prv transport)	This study
LJK3-S	LJK3 Str^r^	This study
**Plasmids**		
F′_8_	transfer direction 17.1 min 16.7 min	[18]
F′_100_	17.7 12.1	[19]
F′_125_	21.4 31.7	[19]
F′_129_	54.0 44.9	[19]
F′_152_	17.2 13.0	[19]
F′_198_	58.6 53.6	[19]

### Transduction and conjugation

Transductions were carried out with P1.kc essentially as described previously [11]. F′-plasmid conjugation was done with cells growing in LB medium to mid-logarithmic phase. Cells were washed, resuspended in a small volume and cross-inoculated on the selection plate. As control, the individual strains were inoculated on the same plate. Cells were grown usually for 16 hours at 37°C and ex-conjugants purified on non-selective plates. F′_100_ and F′_152_ plasmids harboring strains were used for conjugation into *E. coli* LAB65 (Nag^−^) or *E. coli* PS8-1 (RecA^−^ Gal^−^) to exclude the possible occurrence of Hfr strains.

### Radioactive transport assays

For radioactive transport assays bacteria were grown exponentially to about 5×10^8^ cells/ml, harvested and washed three times in 1% NaCl. The cells were resuspended in minimal medium at 25°C to 5×10^8^ cells per ml and tested for specific transport with substrate concentrations of varying range as mentioned in the text; the activities were calculated from the initial uptake rates (0 to 30 sec) and are expressed in nmoles per min per mg of protein [12].

### Determination of extracellular pyruvate

The concentration of extracellular pyruvate was measured enzymatically based on the oxidation of NADH, detected spectrophotometrically at a wavelength of 340 nm using the pyruvate test kit UV-726 from SIGMA according to manufactures instructions.

## Results

### Physiological characterization of pyruvate uptake in *E. coli K-12*

To test whether the uptake of pyruvate was inducible, uptake of [1-^14^C] pyruvate (at 6 µM) was tested in *E. coli* strain K-12/LJ110 [13], after growth in LB medium and in minimal medium supplemented with various carbohydrates. The cells were grown to mid-logarithmic phase for maximal pyruvate uptake activity [8]. Transport activities (Table 2) were lowest after growth on gluconate, LB and glucose medium (1.6 to 2.6 nmol/min×mg protein), intermediate after growth on succinate, glycerol and D-glucitol (4.9 to 5.9 nmol/min×mg protein), and highest after growth on pyruvate (9.4 nmol/min×mg protein). This suggested an inducible pyruvate transport activity, which was also susceptible to catabolite repression. *E. coli* strain K-12/CA8000 [14], and a wild type isolate of *E. coli*, strain EC3132 [11] gave similar results after growth on glycerol and pyruvate, thus indicating that the inducibility is not restricted to our wild type reference strain *E. coli* LJ110 (data not shown).

**Table 2:** Transport activity of *E. coli* LJ110 during growth on various carbohydrates and LB.

Growth substrate	LB	Gluconate	Glucose	Succinate	Glucitol	Glycerol	Pyruvate
Specific activity 1	2.4	1.6	2.6	4.9	5.3	5.9	9.4
% 2	25	6	27	52	56	62	100

1 Specific activity in nmol/min×mg protein;

2 pyruvate set as 100%; mid-log cells grown aerobically at 37°C in LB, or in minimal medium supplemented with (0.2%) carbohydrates, were washed and tested for [1-^14^C] pyruvate uptake (6 µM final concentration) as described in materials and methods. Results are mean averages from at least 3 independent experiments. Standard deviations were less than 10%.

**Figure 1:**
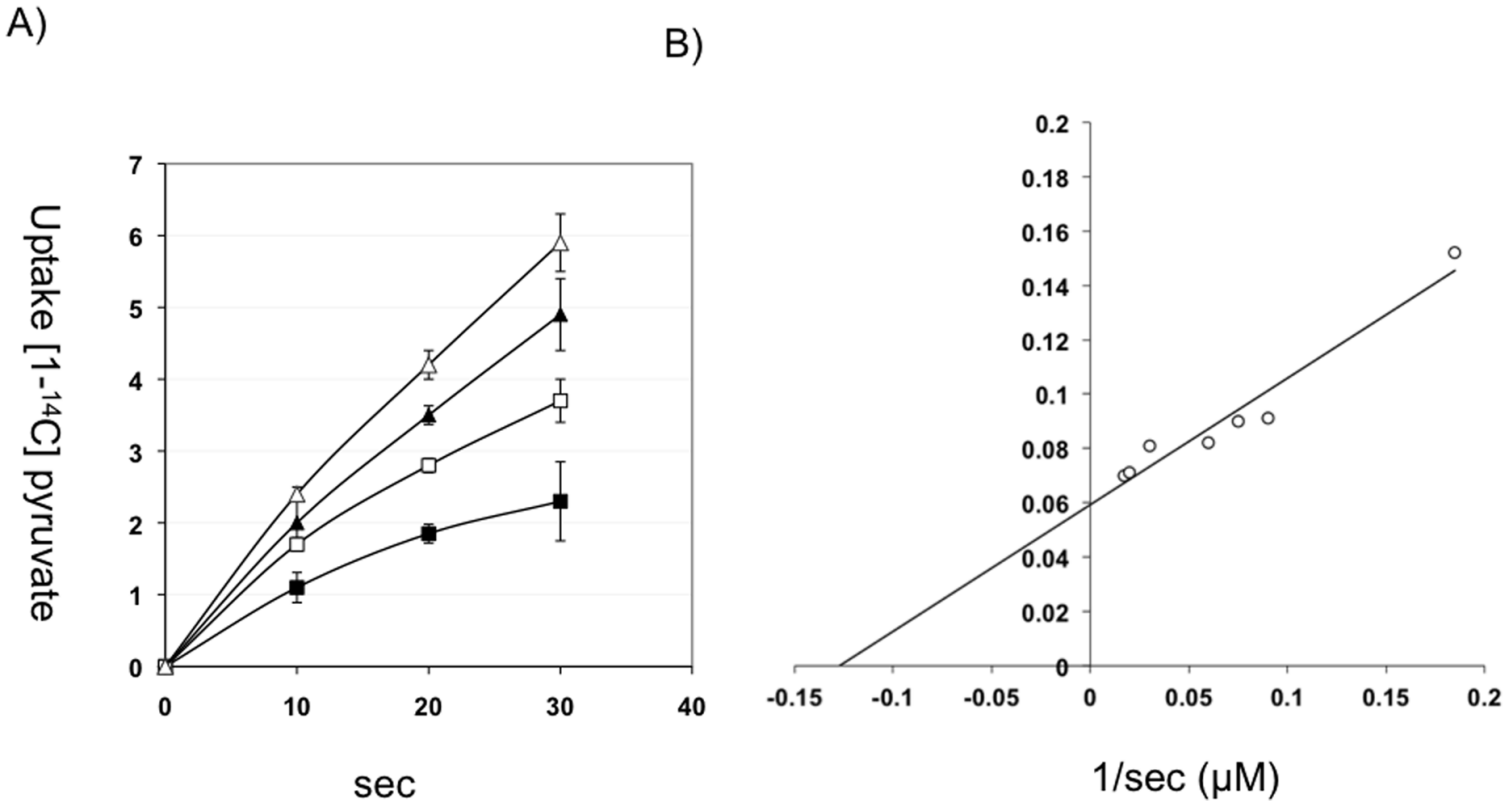
Transport-activity, apparent *K_m_* and *V_max_* values for pyruvate uptake in *E.coli* LJ110. (A) Transport-activity of [1-^14^C] pyruvate uptake determined with four different substrate concentrations: Δ 44 µM, ▴ 26 µM, □ 13 µM, ▪ 5 µM. (n = 3) (B) Double reciprocal plot to determine the apparent *K_m_* value and the corresponding *V_max_* value for LJ110. The initial uptake rates were used to calculate the *K_m_* and *V_max_* at substrate concentrations ranging from 5 µM to 66 µM.

Previous assays of pyruvate transport in *E. coli* were performed either in vesicles or in cells that carried mutations in the enzymes pyruvate dehydrogenase (Pdh) and phosphoenolpyruvate-synthase (PpsA), two main routes for pyruvate metabolism in *E. coli* [8]. In addition, the cells were grown with acetate and glycerol, i.e., pyruvate transport was not induced [8]. In contrast, we used the wild type strain *E. coli* LJ110, to avoid accumulation of intracellular pyruvate, and the cells were tested for pyruvate uptake after growth in minimal medium with pyruvate (0.2%). Uptake activity of [1-^14^C] pyruvate at concentrations ranging from 5 to 44 µM is shown in (Fig. 1A). The apparent *K_m_* value of 7 µM and a *V_max_* of 20 nmol/min×mg protein was calculated from tests with pyruvate concentrations ranging from 5 to 66 µM, and with cells grown to the mid-logarithmic phase, (Fig. 1B). This compares well with previously measured values from the mutant impaired in pyruvate metabolism [8].

### The toxic pyruvate analog 3-FP inhibits pyruvate transport and growth of *E. coli* LJ110

**Figure 2:**
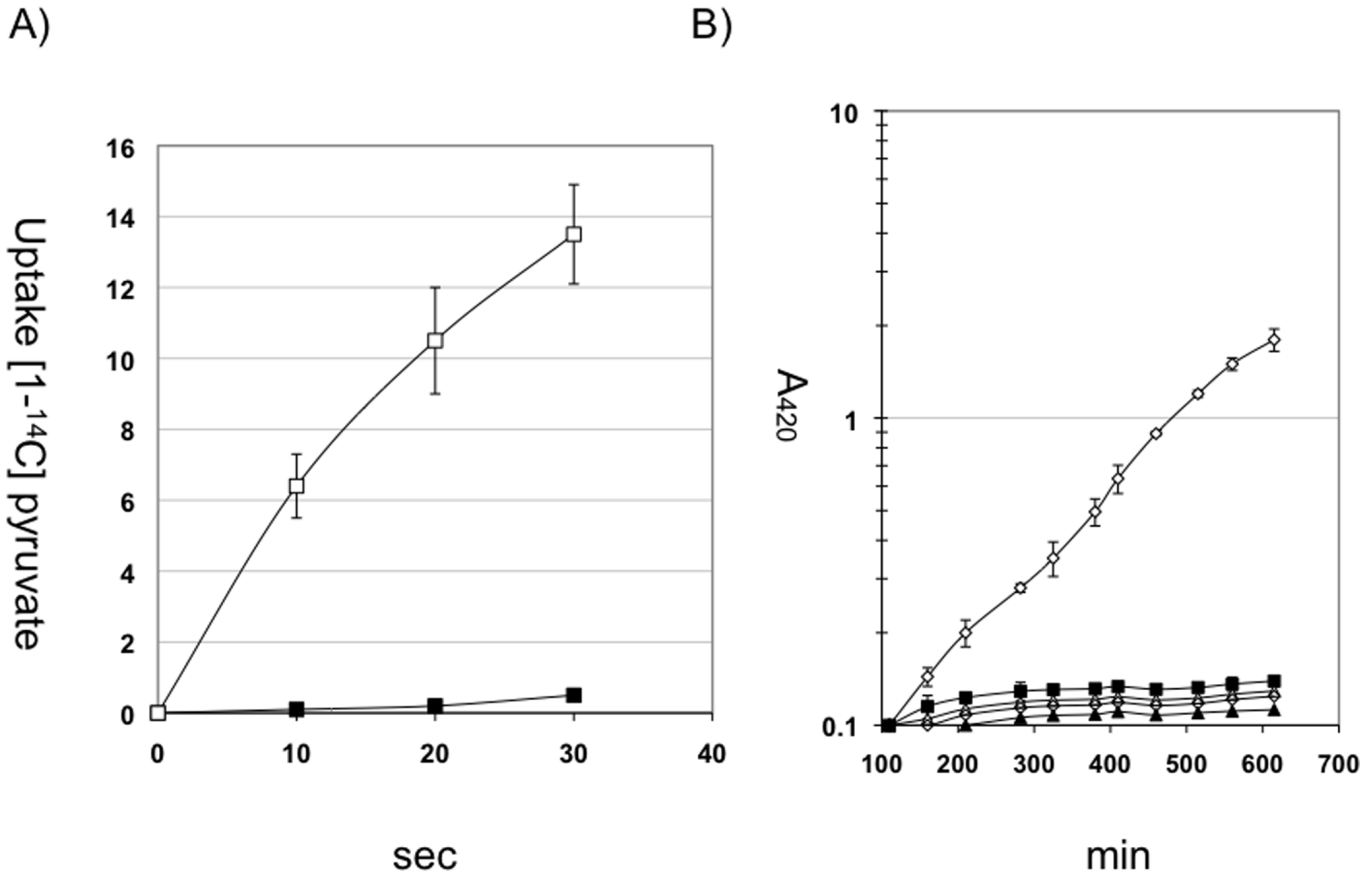
Influence of FP on growth and pyruvate transport-activity on *E. coli* LJ110 (A) Cells were grown in minimal medium plus 0.2% pryruvate. The transport activity was determined with 26 µM [1-^14^C] pyruvate with (▪) and without (□) the addition of 2 mM FP. (n = 3) (B) The growth inhibitory effect of different FP concentrations was determined with cells in the early log phase grown on minimal medium plus 0.2% glucitol. □ 0 mM FP, ▪ 0.1 mM FP, Δ 0.25 mM FP, ◊ 0.5 mM FP, ▴ 1 mM FP. (n = 3)

To further characterize the pyruvate transport properties of strain *E. coli* LJ110, the pyruvate analogue 3-FP was tested as an inhibitor for pyruvate uptake in whole cells. Previous studies demonstrated an inhibition of pyruvate transport in *E. coli* membrane vesicles by 3-FP of about 60% [8]. In cells of LJ110 pre-grown on MM pyruvate, the addition of 2 mM 3-FP inhibited the uptake of 26 µM [1-^14^C] pyruvate nearly completely (Fig. 2A), thus indicating that both substrates may share the same uptake system. We therefore also tested the influence of increasing 3-FP concentrations on the growth of LJ110. Cells were pre-grown in minimal medium with d-glucitol for one generation to partially express pyruvate uptake activity, before 3-FP was added to final concentrations ranging from 0.1 mM to 1 mM. In all samples, the addition of 3-FP instantaneously inhibited growth, maximal inhibition being caused by 1 mM 3-FP or higher (Fig. 2B). Cells growing on minimal medium with succinate, D-fructose, lactose, glycerol or D-mannitol were equally inhibited by 3-FP at 1 mM (data not shown). The transient initial increase in cell density after the addition of 3-FP was completely prevented when the cells were fully pre-induced for pyruvate transport prior to 3-FP addition. Furthermore, the inhibitory effect of 1 mM 3-FP could be neutralized by the addition of a 100-fold excess of pyruvate, while the addition of a 100-fold excess of L-lactate had no protective effect (data not shown). This again argues against an efficient uptake of L-lactate through the pyruvate uptake system, and against uptake of 3-FP through the LldP permease.

### Selection of pyruvate transport negative mutants by means of 3-FP

**Figure 3:**
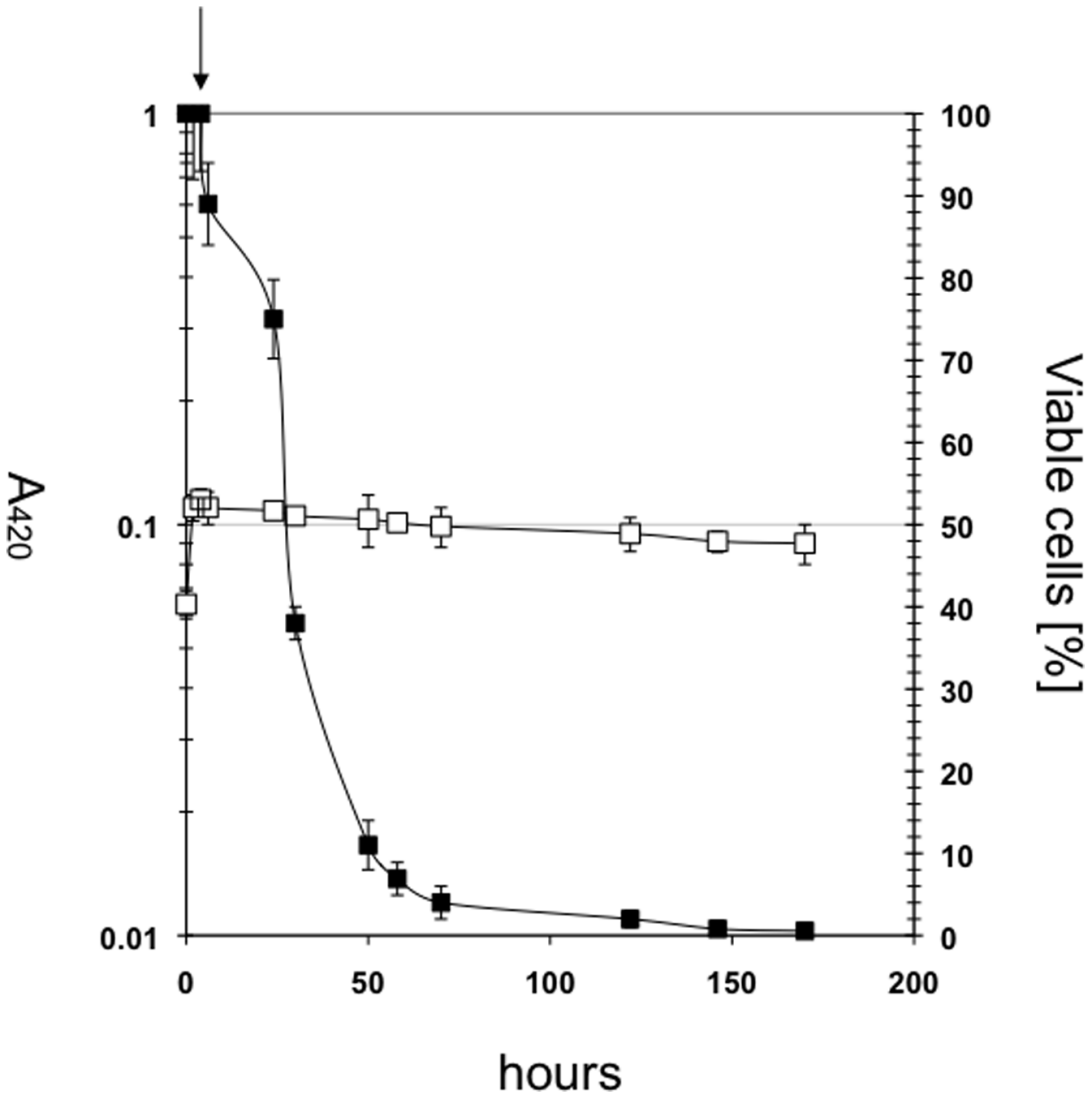
Influence of FP on cell viability of *E. coli* LJ110. The cells were grown in minimal medium plus glycerol (0.2%). Addition of FP (1 mM) is indicated by the arrow. Cell-viability was determined by plating cells at indicated time-points on LB plates. □ A_420_, ▪ viable cells [%].(n = 2)

Similar to Bromo-pyruvate [8], 3-FP could theoretically also be used to isolate pyruvate transport negative mutants. Consequently, cells were incubated in minimal glycerol medium plus 1 mM FP. Subsequently, absorption was measured and cell viability was controlled by serial dilution and plating for cell enumeration (Fig. 3). Cell viability dropped over the time period of 160 h from 100 to about 3%, thus demonstrating the bactericidal effect of 3-FP on the cells. Upon microscopic inspection, cell integrity and cell shape in the treated cultures was unaltered, and no obvious cell lysis could be detected. The bactericidal effect was not caused primarily by the decay of 3-FP and a release of fluorine, since addition of a 100-fold excess of pyruvate allowed outgrowth of the remaining viable bacteria.

To select 3-FP resistant mutants, cells of *E. coli* LJ110 were grown in minimal media with pyruvate. After one doubling time, cells were washed and inoculated in minimal media with D-glucitol and 1 mM 3-FP and incubated aerobically at 37°C. After 30 days the culture became turbid, indicating outgrowth of putative resistant mutants. These were purified on McConkey plates plus glucose, and about 100 colonies were tested for growth on minimal medium agar plates with 20% pyruvate as sole carbon source. Twelve of these were unable to grow overnight on pyruvate, but further incubation for 24 hours led to clones, which showed weak growth. The 12 clones were able to grow either on acetate plus pyruvate and dl-lactate which excludes mutations in the phosphoenolpyruvate-synthase (PpsA) [4] or on succinate which excludes pyruvate dehydrogenase (Pdh) negative mutants [15]. Cells were resistant to 1 mM 3-FP when grown on succinate minimal medium agar plates. One of these, named *E. coli* LJK3, was characterized further.

**Figure 4:**
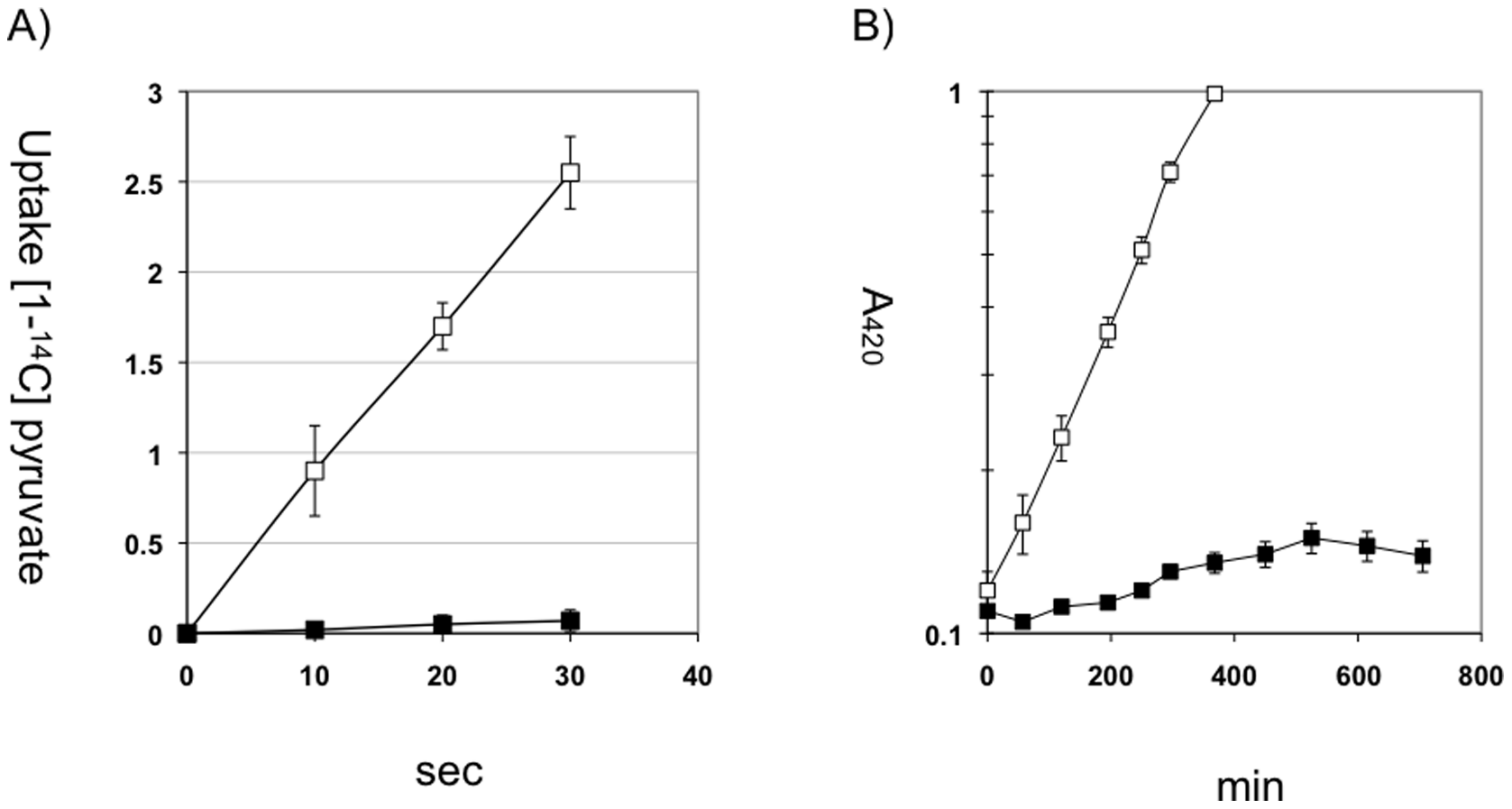
Transport-activity and growth on pyruvate. (A) Pyruvate uptake of *E. coli* LJ110 and LJK3 was measured with [1-^14^C] pyruvate at a final concentration of 6 µM. Cells were grown in minimal medium plus glycerol (0.2%). (n = 3) (B) Cells pre-grown on minimal medium plus glycerol (0.2%) were washed and inoculated in minimal medium plus pyruvate (0.2%) and growth was followed by measurement of absorption at 420 nm. □ LJ110, ▪ LJK3. (n = 3)

Its transport activity and the growth characteristic on pyruvate are shown (Fig. 4 A, B). Cells grown on minimal medium plus glycerol were not able to take up pyruvate. When cells of mutant *E. coli* LJK3 pre-grown on glycerol were washed and inoculated into 0.2% of pyruvate medium, growth was very slow (generation time ≥600 min) and stopped completely after about 300 min. This contrasts the parent *E. coli* LJ110 that grew with a doubling time of 125 min and continued growing beyond 300 min. On minimal medium D-glucitol plus 1 mM 3-FP, growth of *E. coli* LJK3 was somewhat retarded, 200 min as compared to 120 min on D-glucitol alone, indicating a weak inhibition by 3-FP. This weak inhibition and the transient growth on pyruvate possibly indicate some remaining uptake activity.

The transport activity for [1-^14^C] pyruvate (6 µM) of *E. coli* LJK3 grown on minimal medium glycerol was below 0.1 nmol/min per mg protein compared to 5.4 for the wild type, and no induction of transport activity through pre-incubation with pyruvate was possible.

### Isolation and characterization of pyruvate positive derivatives from *E. coli* LJK3

When cells of *E. coli* LJK3 were incubated longer on minimal medium pyruvate plates, colonies formed within three days. Such colonies were purified on LB plates and two, named *E. coli* LJK3R1 and *E. coli* LJK3R2, were further tested. Both grew on pyruvate with slightly reduced doubling times (155 versus 125 min for *E. coli* LJ110) (Table 3). When grown in minimal medium with glycerol, their transport activity was barely detectable, similar to the activity of *E. coli* LJK3. However, and in contrast to *E. coli* LJK3, their activity could be induced by growth on pyruvate to the level of un-induced cells of strain *E. coli* LJ110 after growth on glycerol, but not further (Table 3). When tested as before for 3-FP sensitivity, un-induced cells of *E. coli* LJK3R1 and *E. coli* LJK3R2 could grow in the presence of 3-FP, although with a reduced generation time (330 min compared to 152 min for cells without 3-FP), while pyruvate-induced cells like the wild type stopped growth completely (data not shown). Obviously, both Prv^+^ mutant strains regained an inducible transport system for pyruvate.

**Table 3:** Pyruvate Transport activities of *E. coli* LJK3R1 and LJK3R2.

Strain	LJ110	LJK3	LJK3R1	LJK3R2
Glycerol	5.3	≥0.1	0.5	0.4
Pyruvate	9.4	≥0.1	5.5	5.4

Presented is the specific activity in nmol/min×mg protein; mid-log cells grown aerobic at 37°C in the listed minimal medium supplemented with (0.2%) carbohydrate source were washed and tested for [1-^14^C] pyruvate uptake (6 µM final concentration) as described in materials and methods. Results are mean averages from at least 3 independent experiments. Standard deviations were less than 10%.

### Determination of the chromosomal loci conferring 3-FP resistance

A set of F′- plasmids covering defined parts of the *E. coli* K-12 chromosome were used in an attempt to complement the pyruvate negative phenotype. For selection purposes a spontaneous streptomycin resistant mutant of *E. coli* LJK3, LJK3-S was isolated. *E. coli* LJK3-S showed the same pyruvate negative phenotype as *E. coli* LJK3 (data not shown) and was used for conjugation with F′-plasmid carrying strains. Plasmids F′_152_ and F′_100_ were able to complement the pyruvate negative phenotype. F′_8_ which covers a smaller region from 17.1 min to 16.7 min was not able to complement, narrowing the region for a putative uptake system of pyruvate between 13 and 16.7 min on the *E. coli* K-12 chromosome. Strain *E. coli* LJK3-S/F′_152_ was further investigated for the uptake of pyruvate. Interestingly, the specific transport activity measured with cells grown on minimal medium with glycerol was comparable to cells induced with pyruvate ranging between 4 and 5 nmol/min per mg protein, respectively. There was no difference in the sensitivity against FP between induced and un-induced cells. These results suggest that a constitutively expressed transport system for pyruvate is located between 13 and 16.7 min on the chromosome.

In an attempt to further map the location of the gene responsible for constitutive pyruvate uptake (named hereafter *prvT*), we determined the co-transduction frequencies between the *nag* genes for the utilization of N-acetylglucosamin at 15.1 min, a *kdp*::Tn*10* marker at 15.6 min and *prvT*. We used a P1. lysate of *E. coli* strain LAB65 Nag^−^ *prvT^+^ kdp*::Tn*10* for transduction into *E. coli* LJK3 Nag^+^ *prvT^−^* Kdp^+^. The co-transduction frequency for all three markers was 77%, whereas in 23% only the kdp::Tn*10* marker was transduced. A co-transduction of *prvT* with only one other marker did not occur. This narrows the putative position of *prvT* upstream of *nagA*. A tested transductant showed a similar constitutive pyruvate transport and FP sensitivity as *E. coli* strain LJK3-S/F′_152_ (data not presented).

### Increased pyruvate excretion by *E. coli* LJK3 compared to the wildtype *E. coli* LJ110

**Figure 5:**
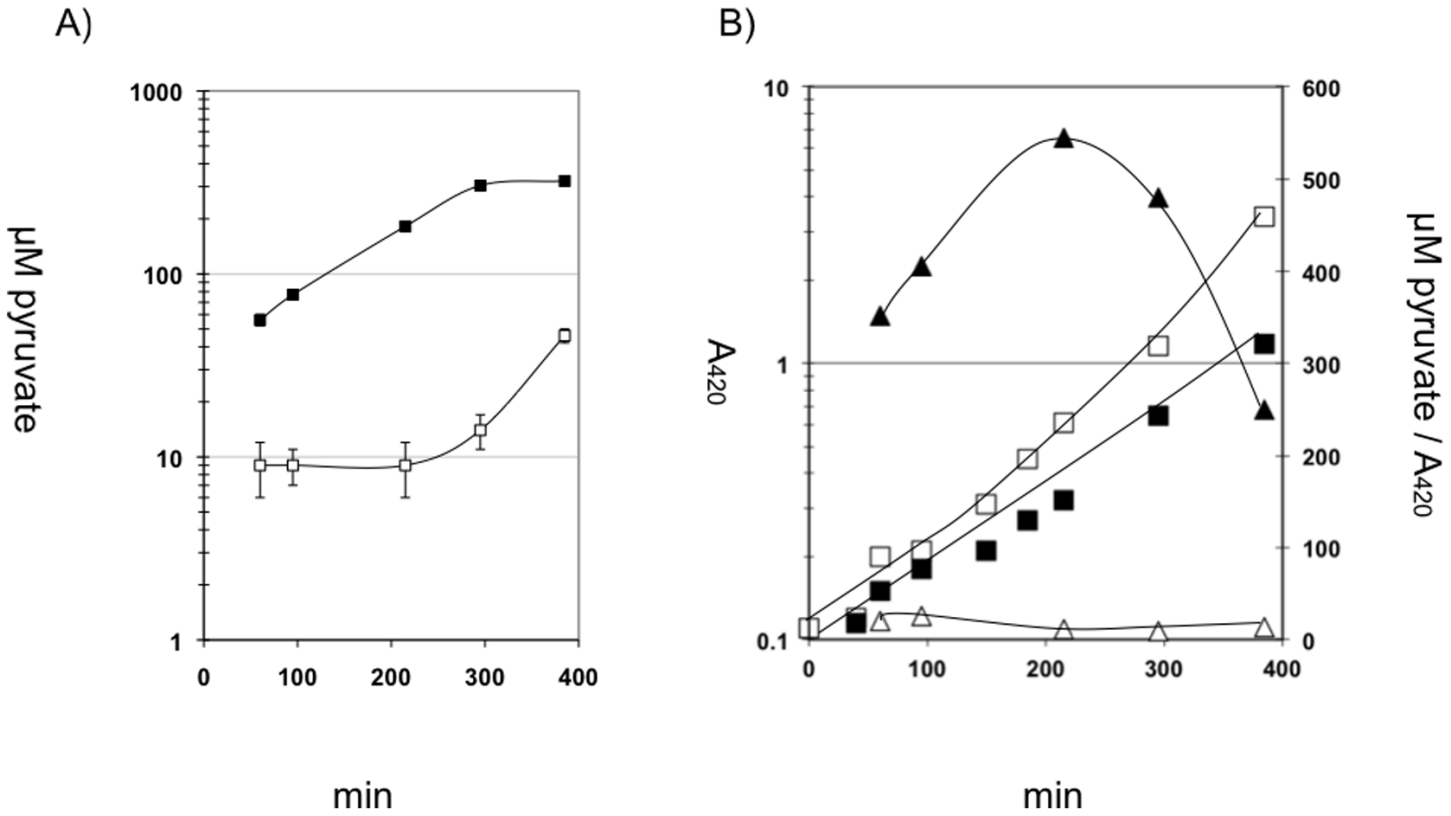
Concentration of extracellular pyruvate during growth of *E. coli* LJK3 under potassium limiting conditions. (A) Pyruvate concentration in µM; □ LJ110, ▪ LJK3. (n = 3) (B) Pyruvate concentration as a function of cell-density. Cells were pre-grown in minimal medium plus glucose and 115 mM potassium (0.2%) and inoculated in medium plus glucose and no potassium (0.4%). Extracellular pyruvate was measured enzymatically as described in materials and methods. A_420_ = □ LJ110, ▪ LJK3; µM pyruvate/A_420_ = Δ LJ110, ▴ LJK3.

Under conditions, where carbohydrate influx exceeds carbohydrate metabolism, *E. coli* is able to excrete intracellular pyruvate into the medium, in particular under the conditions of potassium limitation [3]. Since *E. coli* LJK3 is unable to take up pyruvate efficiently, we tested whether the mutant was still able to excrete pyruvate. Strains *E. coli* LJ110 and *E. coli* LJK3 were grown under potassium-limiting conditions with an excess of 0.4% glucose. (Fig. 5 A) During the 400 min growth period, strain *E. coli* LJ110 produced from 10 to 50 µM extracellular pyruvate, mostly during the end of growth, while *E. coli* LJK3 began immediately to accumulate from 60 to 300 µM extracellular pyruvate. The difference is best seen when the extracellular pyruvate is plotted as a function of cell-density (Fig. 5B). Strain *E. coli* LJ110 exhibited a nearly constant level of extracellular pyruvate versus growth rate, probably reflecting equilibrium between excretion and uptake rates. In contrast, the extracellular pyruvate concentration of *E. coli* LJK3 peaked after 200 min. Experiments under potassium saturation conditions (115 mM) gave similar results. Interestingly, strains *E. coli* LJK3R1 and *E. coli* LJK3R2, lacking the constitutive uptake system, showed extracellular pyruvate levels comparable to strain *E. coli* LJ110 containing both uptake systems (data not presented). These results indicate that uptake and excretion of pyruvate might also use different transport systems.

## Discussion

Pyruvate, an important metabolite of central metabolism, can be taken up and excreted in *E. coli*. In general, the knowledge about pyruvate transport in bacteria is scarce, basically restricted to the identification of the secondary transport proteins MctC in *Corynebacterium glutamicum* [16] and MctP in *Rhizobium leguminosarum* [17]. The MctC transporter depends on the electrochemical proton potential, and its specificity is restricted to acetate and propionate, with a low affinity for pyruvate [16]. In contrast, the MctP permease belongs to the class of sodium solute transporters with preference for C_3_-monocarboxylates, including lactate (K_m_ value 4.4 µM) and pyruvate (K_m_ value 3.8 µM) [17].

This is contrary to *E. coli*, in which the major uptake of pyruvate and lactate depends on separate transport systems as first described by Kornberg and Smith by means of growth experiments [4]. This is in agreement with our observation that a deletion mutant of the L-lactate permease could grow on pyruvate with a similar doubling-time as the wild type (data not shown), making it unlikely that pyruvate and L-lactate are transported through the same uptake system in *E. coli*.

Subsequently, pyruvate uptake in whole cells was shown to be inhibited by respiratory chain inhibitors and uncouplers up to 95%, except for arsenate, and to require an artificial electron donor system in membrane vesicles [8]. This argues for at least one major pyruvate uptake system of the class proton gradient dependent secondary transport system in *E. coli*.

Earlier characterizations of pyruvate transport in *E. coli* with a Pdh^−^ PpsA^−^ pyruvate negative mutant uninduced for pyruvate transport [8] determined a K_m_ value for pyruvate at 20 µM, comparable to our results obtained with the fully induced wild type strain *E. coli* LJ110.

Although the existence of a specific pyruvate transporter in *E. coli* has been assumed for some time, only one gene locus designated Usp relevant for pyruvate uptake had been identified mapped near 64 min of the *E. coli* K-12 chromosome [4]. In contrast, transductants from a P1-transduction involving genes *kdp* and *nag* near 15 min of the gene map had regained a constitutive pyruvate transport activity conferred by a gene locus, called PrvT by us (data not presented). This suggest that the Usp system is an inducible pyruvate transport system, and that a new system, named PrvT by us, is expressed constitutively. This system, which was expressed under non-inducing conditions corresponds most likely to the system analyzed by Lang *et al.* [8], i.e. to a proton gradient dependent secondary transport system. Both systems seem to be controlled by carbon catabolite repression, and have a very similar substrate specificity and affinity as observed in PrvT^+^ Usp^−^, and in PrvT^−^ Usp^+^ strains.

Finally, the excretion of pyruvate into the medium, combined with the lack of pyruvate re-uptake ability in the PrvT^−^ Usp^−^ double mutant *E. coli* LJK3, argues for an additional transport system responsible for pyruvate excretion. It remains to be shown to which class of transporters the three systems belong. In analogy to other bacteria which take up, excrete and re-use central metabolites, in particular of the mono-, di- and tri-carboxylates class, these can be expected to be either binding protein dependent primary transporters, and proton- or sodium-gradient dependent secondary transporters, or hybrids thereof.

## References

[1] Postma PW, Lengeler JW, Jacobson GR (1993) Phosphoenolpyruvate:carbohydrate phosphotransferase systems of bacteria. Microbiol Rev 57: 543–594. 8246840 10.1128/mr.57.3.543-594.1993 PMC372926

[2] Boeck A, Sawers G (1996) Fermentation. In: Neidhardt FC, Curtiss RI, Ingraham JL, Lin ECC, Low KB et al., *Escherichai coli* and *Salmonella*. Washington, DC: ASM. 217–261.

[3] Kodaki T, Murakami H, Taguchi M, Izui K, Katsuki H (1981) Stringent control of intermediary metabolism in *Escherichia coli*: pyruvate excretion by cells grown on succinate. J Biochem 90: 1437–1444. 7040357 10.1093/oxfordjournals.jbchem.a133610

[4] Kornberg HL, Smith J (1967) Genetic control of the uptake of pyruvate by *Escherichia coli* . Biochem Biophys Acta 148: 591–592. 4864638 10.1016/0304-4165(67)90167-5

[5] Matin A, Konings WN (1973) Transport of lactate and succinate by membrane vesicles of *Escherichia coli*, *Bacillus subtilis* and a pseudomonas species. Eur J Biochem 34: 58–67. 4349657 10.1111/j.1432-1033.1973.tb02728.x

[6] Dong JM, Taylor JS, Latour DJ, Iuchi S, Lin EC (1993) Three overlapping *lct* genes involved in L-lactate utilization by *Escherichia coli* . J Bacteriol 175: 6671–6678. 8407843 10.1128/jb.175.20.6671-6678.1993 PMC206779

[7] Nunez MF, Kwon O, Wilson TH, Aguilar J, Baldoma L, et al (2002) Transport of L-Lactate, D-Lactate, and glycolate by the LldP and GlcA membrane carriers of *Escherichia coli* . Biochem Biophys Res Commun 290: 824–829. 11785976 10.1006/bbrc.2001.6255

[8] Lang VJ, Leystra-Lantz C, Cook RA (1987) Characterization of the specific pyruvate transport system in *Escherichia coli* K-12. J Bacteriol 169: 380–385. 3025181 10.1128/jb.169.1.380-385.1987 PMC211778

[9] Tanaka S, Lerner SA, Lin EC (1967) Replacement of a phosphoenolpyruvate-dependent phosphotransferase by a nicotinamide adenine dinucleotide-linked dehydrogenase for the utilization of mannitol. J Bacteriol 93: 642–648. 4289962 10.1128/jb.93.2.642-648.1967 PMC276489

[10] Epstein W, Kim BS (1971) Potassium transport loci in *Escherichia coli* K-12. J Bacteriol 108: 639–644. 4942756 10.1128/jb.108.2.639-644.1971 PMC247121

[11] Bockmann J, Heuel H, Lengeler JW (1992) Characterization of a chromosomally encoded, non-PTS metabolic pathway for sucrose utilization in *Escherichia coli* EC3132. Mol Gen Genet 235: 22–32. 1435727 10.1007/BF00286177

[12] Schmid K, Schupfner M, Schmitt R (1982) Plasmid-mediated uptake and metabolism of sucrose by *Escherichia coli* K-12. J Bacteriol 151: 68–76. 6211435 10.1128/jb.151.1.68-76.1982 PMC220195

[13] Zeppenfeld T, Larisch C, Lengeler JW, Jahreis K (2000) Glucose transporter mutants of *Escherichia coli* K-12 with changes in substrate recognition of IICB(Glc) and induction behavior of the *ptsG* gene. J Bacteriol 182: 4443–4452. 10913077 10.1128/jb.182.16.4443-4452.2000 PMC94615

[14] Brickman E, Soll L, Beckwith J (1973) Genetic characterization of mutations which affect catabolite-sensitive operons in *Escherichia coli*, including deletions of the gene for adenyl cyclase. J Bacteriol 116: 582–587. 4583241 10.1128/jb.116.2.582-587.1973 PMC285421

[15] Guest JR, Creaghan IT (1973) Gene-protein relationship of the alpha-keto acid dehydrogenase complexes of *Escherichia coli* K-12: isolation and characterization of lipoamide dehydrogenase mutants. J Gen Microbiol 75: 197–210. 4578971 10.1099/00221287-75-1-197

[16] Jolkver E, Emer D, Ballan S, Kramer R, Eikmanns BJ, et al (2009) Identification and characterization of a bacterial transport system for the uptake of pyruvate, propionate, and acetate in *Corynebacterium glutamicum* . J Bacteriol 191: 940–948. 19028892 10.1128/JB.01155-08 PMC2632059

[17] Hosie AH, Allaway D, Poole PS (2002) A monocarboxylate permease of *Rhizobium leguminosarum* is the first member of a new subfamily of transporters. J Bacteriol 184: 5436–5448. 12218032 10.1128/JB.184.19.5436-5448.2002 PMC135354

[18] Fiethen L, Starlinger P (1970) Mutations in the galactose-operator. Mol Gen Genet 108: 322–330. 4924696 10.1007/BF00267769

[19] Low KB (1972) *Escherichia coli* K-12 F-prime factors, old and new. Bacteriol Rev 36: 587–607. 4568765 10.1128/br.36.4.587-607.1972 PMC408333

